# Child Relativized Minimality and Grammaticality Judgement

**DOI:** 10.3389/fpsyg.2020.00105

**Published:** 2020-02-07

**Authors:** Anna Gavarró

**Affiliations:** Departament de Filologia Catalana, Centre de Lingüística Teòrica, Universitat Autònoma de Barcelona, Barcelona, Spain

**Keywords:** grammaticality judgement, processing, child grammar, object relatives, Relativized Minimality violations, Catalan

## Abstract

Grammaticality judgements are the fundamental experimental source of generative linguistic theory. They may be difficult to elicit, especially in some populations, but generally they inform us neatly about what the grammar licenses or, on the contrary, bans. On the other hand, acceptability is multifactorial and therefore, unlike grammaticality judgement, can be quantified. In this paper I consider a particular empirical domain, that of Relativized Minimality (RM) in acquisition, and its relation to the dichotomy between grammaticality and acceptability. Friedmann et al. (2009) argued that children hold a stricter version of RM than adults. In particular, children would require a disjoint feature specification, not just a distinct feature specification, between target and intervener. The literature shows asymmetries in comprehension of subject and object relative clauses in various languages which fulfill the predictions of child RM. Variation between adults and children might be expected not only in production and comprehension, but also in grammaticality judgement. If so, children would be predicted to reject object relatives as well as the classic RM violations. Alternatively, if child RM is a processing effect, the prediction is that children would be able to tease apart object relative clauses from RM violations under favorable processing conditions. The question I address is: do children assimilate RM violations and object relative clauses? Grammaticality judgement should provide an answer to this question. In this paper I present an experiment targeting grammaticality judgement for object relatives and RM violations and report preliminary results for a group of 6-year-old Catalan-speaking children showing that object relatives and RM violations are not judged in a parallel fashion, since RM violations are rejected more often than object relatives.

## Introduction

The literature on language acquisition has attested an asymmetry in the comprehension and production of relative clauses, object relative clauses lagging behind subject relative clauses (see, for English, Brown, 1971; De Villiers et al., 1979; for French, Labelle, 1990; for Portuguese, Corrêa, 1995; for Spanish, Pérez-Leroux, 1995; for German, Adani et al., 2013; for Italian, Contemori and Belletti, 2014, etc.). Friedmann et al. (2009) proposed a new analysis for this well-known asymmetry: subject and object relative clauses differ in the position from which the wh- constituent moves, and they argued that children apply a stricter constraint on A' movement than adults that renders object relatives (under specific conditions) difficult for them. In this report I explore a prediction of Friedmann et al.'s hypothesis if one assumes that this stricter constraint on movement constitutes a truly grammatical constraint (as opposed to the result of a processing limitation): grammaticality judgement, then, should yield the same pattern as production and comprehension.

The report is organized as follows: in this section I detail Friedmann et al.'s hypothesis and state the prediction to test. In section A Grammaticality Judgement Task, I motivate the experimental design and give details about the experimental items, procedure and participants of the pilot study. In section Results, I present the results, and in section Discussion, I consider them against the background literature.

Following the approach of Relativized Minimality (RM) (Rizzi, 1990) to constraint movement, in a configuration like (1), X and Y fail to relate if Z, the intervener, which is structurally closer to X, can act as its antecedent because of its featural configuration.


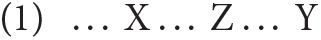


The effect of RM can be illustrated with a classic example such as (2), in which movement of *how* is blocked by the intervening interrogative *who*.


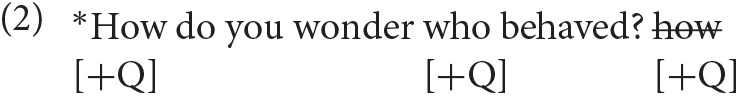


There is no RM violation in a subject relative clause (3), nor in an object relative clause (4).


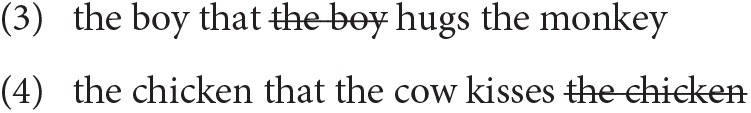


Both are well-formed for adults; however, in Friedmann et al.'s (2009) analysis, for children the subject *the cow* acts as an intervener in (4); there is no possible intervener in (3)^1^. This is argued to be the source of children's delay with object relatives. The configuration in (1) can be instantiated as in (5) [(29) in (Friedmann et al., 2009), p. 84].


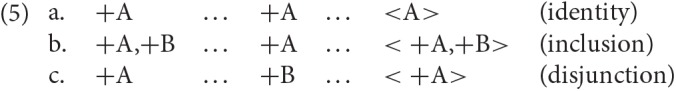


The example in (2) falls under the case of (5a) and is therefore ill-formed for children and adults alike. When B is featurally distinct from A, as in (5c), the resulting sentence is licensed in both child and adult grammar. Differences only emerge with (5b), where the potential intervener, +A, is characterized by a featural configuration that is a subset of the featural configuration of the antecedent +A+B. This corresponds to the configuration underlying object relatives like (4):


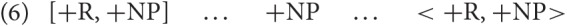


Adult grammar licenses (6), but child grammar treats it as a violation of (a stronger version of) RM. In Friedmann et al.'s (2009, p. 85) words, “a configuration [like that in (6)] is disallowed as it violates the ‘strong' RM requiring featural disjointness.” If object relatives^2^ are assimilated to RM violations in child grammar, the prediction is then that children will judge them as equally ill-formed in a grammaticality judgement task. This prediction is put to test in the experiment described in the next section.

(5) does not exhaust all possible configurations. In later work on featural RM effects in weak island environments, Villata et al. (2016) consider the configurations in (7).


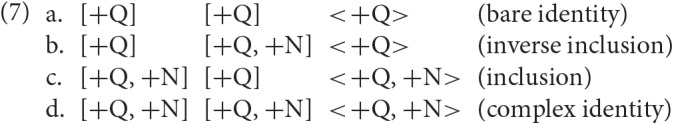


In (5) inverse inclusion (7b) was not considered, and bare identity (7a) and complex identity (7d) fell under identity. Inverse inclusion is exemplified in (8a), complex identity in (8b), both of them examples with intervention (taken from Villata et al., 2016, p. 81).


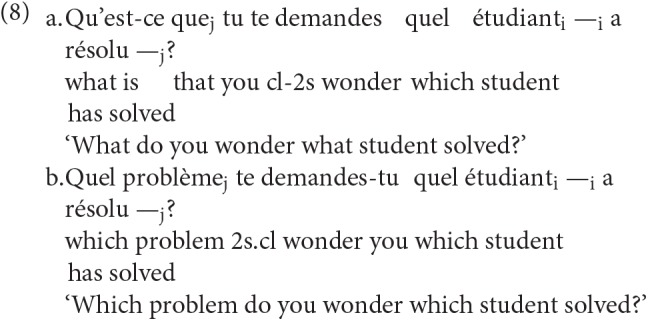


In this paper we focus on the configurations initially considered in Friedmann et al. (2009) and the subsequent research on language acquisition.

## A Grammaticality Judgement Task

The experiment designed is a grammaticality judgement task^3^. Young children experience some difficulty in producing grammaticality judgements, possibly because of the inability of the experimenter to transmit what the task is about, and because the task requires some metalinguistic awareness. For that reason, the children recruited were in the age range of 5–7 years and not younger.

### Materials and Methods

Three sentence types were tested in Catalan: (i) object relative clauses, (ii) long distance wh- questions, and (iii) ungrammatical wh- questions involving RM violations. It is worth stressing that the RM violations in the experiment were ill-formed and not just degraded, as some weak island violations may be—see examples (9), taken from Villata et al. (2016) and the examples in Rizzi (1990), as well as the discussion of gradations of acceptability also in relation to RM in Rizzi (2018).


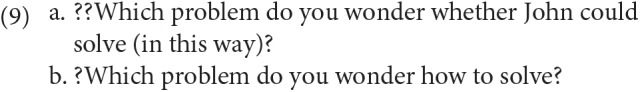


The objective relative in (10) instantiates (5b), the long-distance wh- question in (11) is an instance of (5c), and the wh- question involving a RM violation in (12) instantiates (5a). The featural configuration in (10) is such that the head of the relative clause bears the features [+R,+N], and the intervening DP the feature [+N] (as assumed in Friedmann et al., 2009). The featural configuration of the wh- questions exemplified in (11) is assumed to be [+Q] for the wh- elements involved. Likewise in the ungrammatical question exemplified in (12).


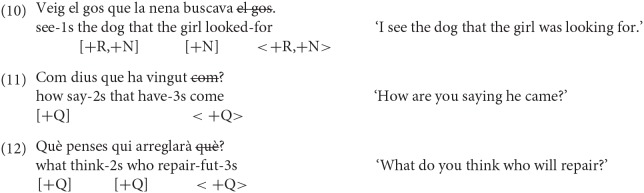


In the well-formed wh- questions, two of the experimental sentences contained *dir* “say” as verb selecting the embedded clause and six contained the verb *pensar* “think/wonder”; the same verbs (and in the same proportion) were used in the ungrammatical RM items. The wh- words used were all bare wh- elements, including *què* “what,” *qui* “who,” *com* “how” and *quan* “when.” Since sentences were produced out of context and, furthermore, no complex wh- phrase was used, the effect of D-linking was excluded. In the wh- questions, the wh- element corresponded to an argument or an adjunct of the embedded clause, either because it was a direct object of the embedded verb, or because, as adjuncts, *quan* “when” and *com* “how” would more naturally modify the embedded clause (as in *When do you think you will go?*). The same is true of the RM items: *què* “what” could only be an argument of the embedded clause; in the remaining cases with *com* “how” the adjunct would most naturally modify the embedded verb, *venir* “come over” or *portar-se* “behave,” and in this last case it was selected by the verb. In the wh- questions and RM items no overt DP intervened between the wh- elements (subjects were null pronouns except in one case in which the overt subject was postverbal and, therefore, lower in the structure).

Each of the three experimental conditions was exemplified by 6 items, and so the total number of test items was 18 (a complete list appears in the Annex). Items were between 7 and 11 syllables long and were presented in pseudorandom order. Of the 18 items, only 6 were ungrammatical for the adult speakers; should children find object relatives ungrammatical, then 12 out of the 18 items would be rejected.

If children assimilate identity configurations (5a) and inclusion configurations (5b), the prediction is that they will perform equally with the two. This is what the literature on child RM has argued: children fail with object relatives when the configuration is that seen in (5b); subject relatives do not give rise to such a configuration, and the subject/object asymmetry follows^4^. A second prediction, not stated by Friedmann et al., is that, if the assimilation of (5b) to (5a) is operative, children will judge instances of (5b) as bad as instances of (5a). This is the rationale of the experiment.

An anonymous reviewer points out that the comparison between object relative clauses and wh- extraction is far from perfect, since these two constructions have been shown to be quite different, so that, for example, in English, Preposition stranding is favored in indirect object wh- questions, but pied-piping is preferred in indirect object relative clauses (Bianchi and Chesi, 2015); in a cross-linguistic study, Sprouse et al. (2016) show that island effects are different between relative clauses and wh- dependencies (in English, adjunct relative clauses do not show island effects, while adjunct wh- dependencies do; Italian does not exhibit subject island effects in relative clauses, but it does in wh- extraction). The reviewer suggests that a better design would therefore include only wh- questions; this remains for future research.

### Participants

The children who participated in the study were native speakers of Central Catalan from the extended metropolitan area of Barcelona. Twenty-five children were tested, but three were excluded because they failed to understand the task. The remaining 22 children were in the age range of 5;05,20 to 7;04,27 (mean age: 6;05). Five adults took part in the experiment as a control group.

The guidelines of the Declaration of Helsinki on human experimentation were enforced during the whole procedure and the experiment was approved by the ethics committee of the UAB (CEEAH evaluation number 4,856).

### Procedure

The experiment was carried out individually in a quiet classroom of the children's school. It involved two experimenters, one manipulating a dog puppet and uttering the target sentences, the other introducing the task and questioning the child. The child was told that the puppet was learning to speak but sometimes said things that didn't sound right and so the child would be asked if the sentences s/he heard uttered by the puppet sounded right. The experimental phase was preceded by a training phase consisting of at least two items, one grammatical, another ungrammatical (*Tinc molta gana* “I am very hungry” vs. ^*^Molta tinc gana “Very I am hungry”); if necessary, the training phase included more items. Positive feedback was given to the child in the experimental phase irrespective of his/her answers. The task took around 15 min. Adults were tested individually on the university campus.

The answers of all participants were recorded on an answer sheet by the second experimenter and then transcribed into an RStudio file.

## Results

Adults performed as expected: they rejected all RM violations and accepted all grammatical long- distance interrogatives and object relatives.

**Figure 1:**
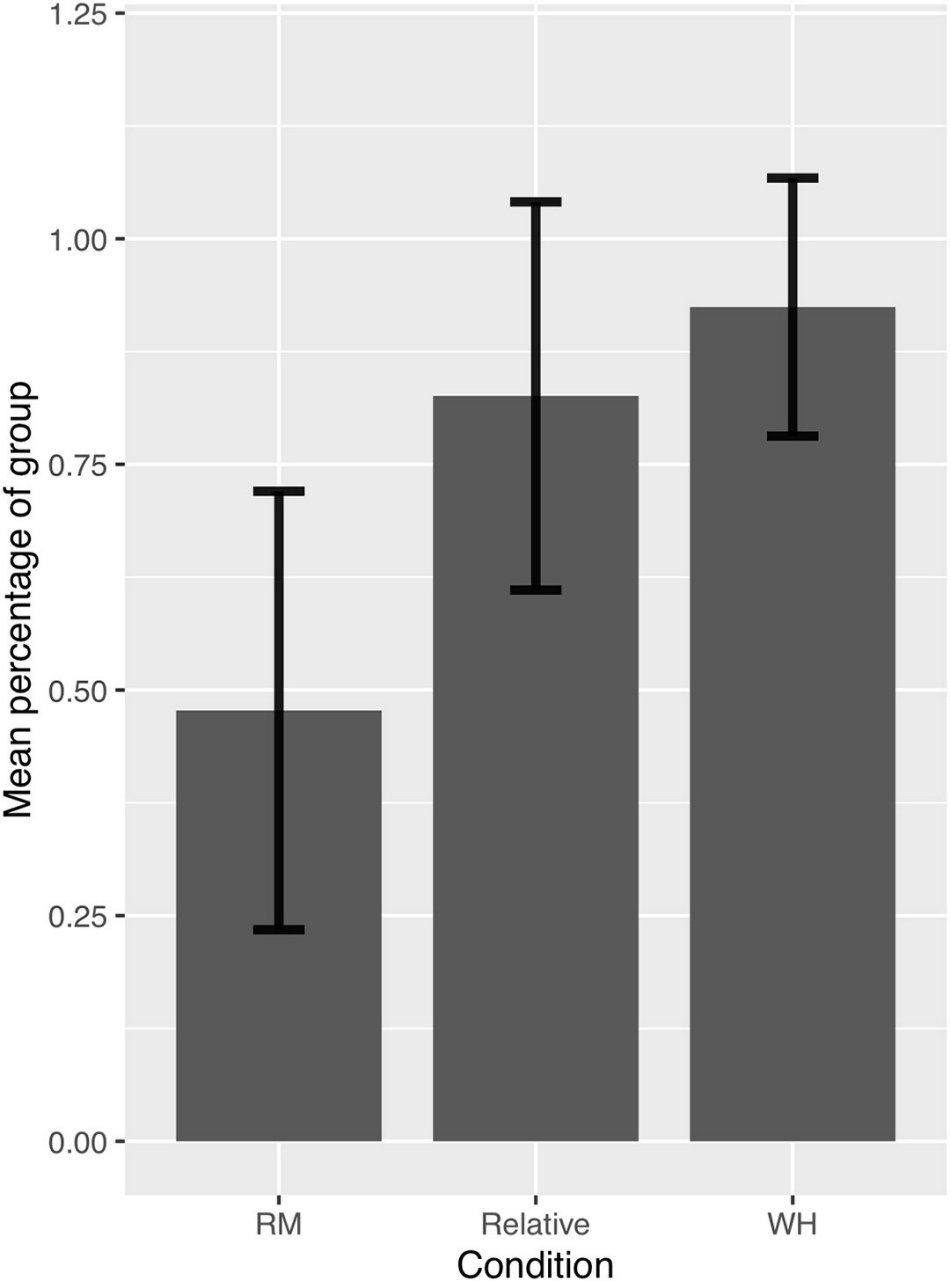
Acceptance rate of the three sentence types, children.

The total number of answers provided by the children was 396 (18 × 22), 132 per condition. The data set is freely available at https://ddd.uab.cat/record/215041. Children performed as shown in Figure 1 and Table 1, representing mean acceptance rate, standard deviation and the five number summary (order statistics) Minimum, Q1, Median, Q3, and Maximum.

**Table 1:** Acceptance of the three sentence types, Mean, SD, and order statistics.

**Stype**	**Data**	**Mean**	**SD**	**Minimum**	**Q1**	**Median**	**Q3**	**Maximum**
Relative	22	0.826	0.215	0.167	0.667	0.833	1.000	1.000
RM	22	0.477	0.243	0.000	0.333	0.417	0.667	0.833
Wh-	22	0.924	0.143	0.000	0.875	1.000	1.000	1.000

**Figure 2:**
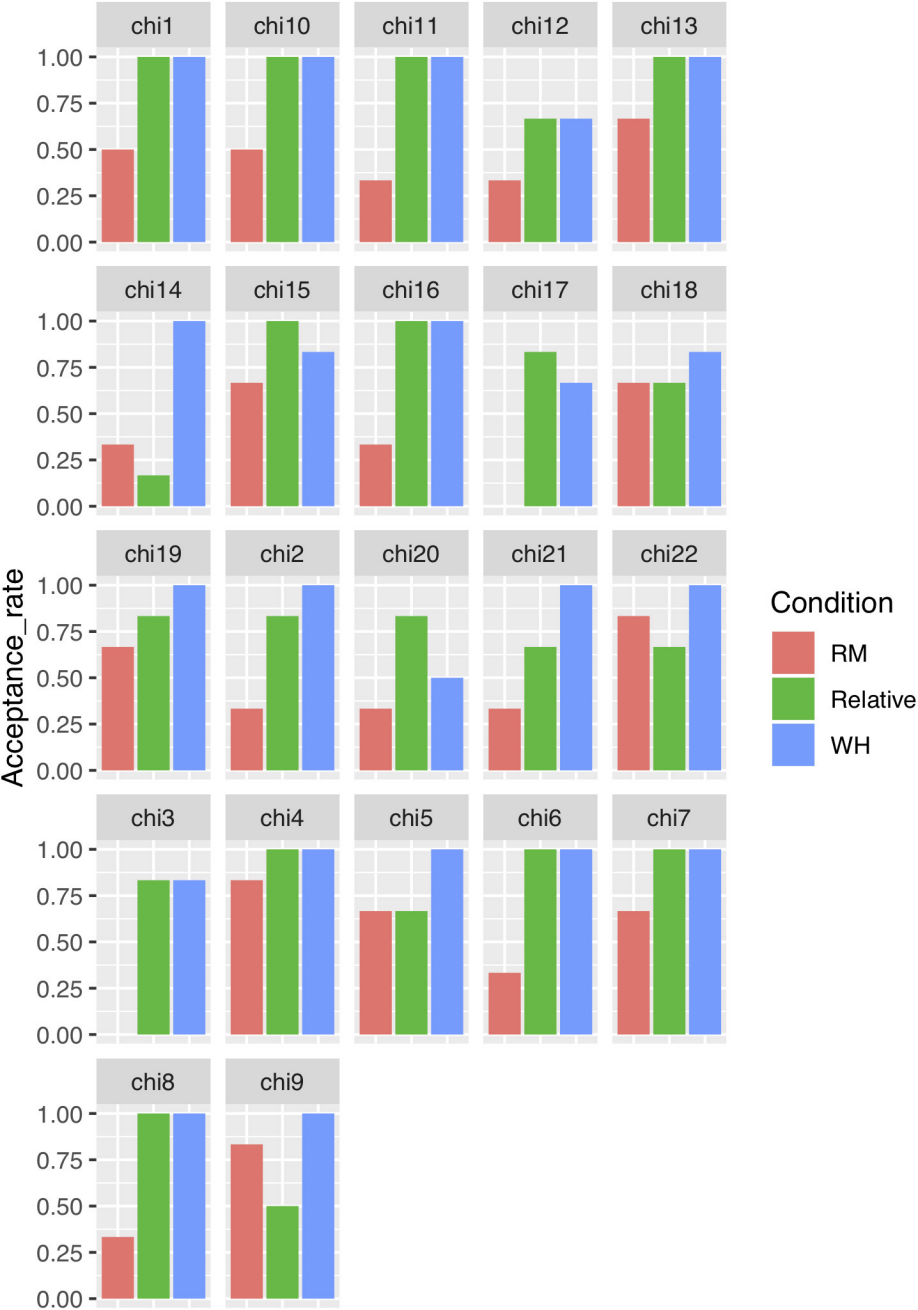
Individual results.

If we turn to individual results, all the children rejected at least one RM violation, while 10 children accepted all object relative clauses. Two children judged these two sentence types identically; three more children judged RM violations better than object relatives. The remaining 17 children accepted object relatives more often than they accepted RM violations, tending toward the adult pattern. Individual results appear in Figure 2.

Even though few children took part in the experiment, and it would be desirable to run it with more participants, some statistical analysis was undertaken. A Generalized Linear Mixed Model was used to model the number of acceptances by sentence type as a binomial response, taking into account repeated measures from each participant. The statistical analysis was obtained using R (R Core Team, 2019).

**Figure 3:**
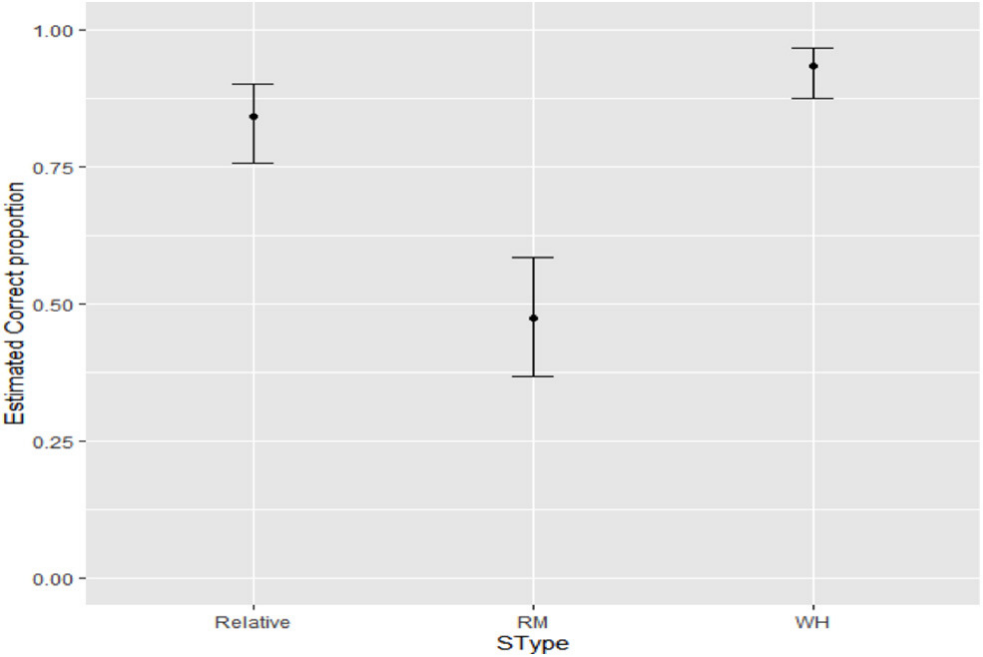
Estimated acceptance of the three sentence types.

Statistically significant differences were found as an effect of Sentence Type (*F*-Value = 63.19; *p*_value < 0.0001). For the RM (ungrammatical) items, the percentage of estimated acceptance responses was 47.48% (CI_95%_ = [36.8%, 58.4%]). For the object relative items, the percentage of estimated acceptance responses was 84.22% (CI_95%_ = [75.59%, 90.19%]). For wh- questions, the percentage of estimated acceptance responses was 93.47% (CI_95%_ =[87.4%, 96.72%]). These results are represented in Figure 3.

Pairwise comparisons of the three sentence types were all significant. There were statistically significant differences between object relatives and RM (z-ratio = 5.8; *p*_value < 0.0001), with higher acceptance of object relatives than RM violations (OR = 5.9, i.e., the odds ratio of acceptance of object relatives was 5.9 times the odds of acceptance of RM violations). There were marginal statistically significant differences between object relatives and wh- questions (z-ratio = −2.4; *p*_value = 0.0424), with higher acceptance of wh- questions (OR = 0.37, i.e., the odds ratio of acceptance of wh- questions was 1/0.37 ≈ 2.68 times the odds for object relatives). Finally, there were statistically significant differences between RM violations and wh- questions (z-ratio = −7.05; *p*_value < 0.0001), with wh- questions being accepted more often than RM violations (OR = 0.0632, i.e., the odds ratio of acceptance of wh- questions was 1/0.0632 ≈ 15.82 times the odds for the RM items).

The results so far show that object relatives and RM violations did not pattern alike for children: children accepted object relatives at much higher rates than RM violations. Rather, object relatives tended to pattern with long-distance wh- questions, as in adult judgements. However, there is a difference in the acceptance rates of object relatives and wh- questions in the judgements of children that is not found in the judgements of adults, albeit the difference is smaller than between any of these two grammatical sentence types and the ungrammatical RM sentences.

These results are tentative; however, with the sample here the hypothesis that object relatives and RM violations are judged in the same way by children cannot be upheld.

## Discussion

In this section, I discuss the results in two respects: first, I consider age and performance in other tasks which, by hypothesis, relate to the one here; second, I go back to the question that motivated this study, namely, does child RM stem from a property of child grammar, defining grammaticality, or does it stem from processing limitations?

First let us consider the results with respect to age. In future research more children and from a wider age range should be tested; with the current sample, the five children who could be considered to conform to the parallel performance in RM violations and object relatives were not amongst the youngest, and performance appears to bear no relation to age (within the limited age span here).

Notice that the children in this study were slightly older than the Hebrew-speaking children in Friedmann et al. (2009), who were in the age range of 3;07 to 5;0. Other studies, however, show that delay in the comprehension of object relatives extends beyond age 5;0. In a study of the acquisition of relative clauses in Catalan, Gavarró et al. (2012), on the basis of a picture identification task, found that the comprehension of object relatives was delayed when compared to the comprehension of subject relative clauses. Production (elicitation based on Novogrodsky and Friedmann's, 2006 method) yielded very similar results [see Table 2, which summarizes the results of the two experiments, administered to 21 children (comprehension) and 20 children (production)].

**Table 2:** Subject and object relative clause comprehension and production, Catalan (Gavarró et al., 2012, p. 194).

	**Subject relatives**		**Object relatives^a^**	
**Comprehension**				
4;06–5;06 (Mean 4;11,06)	64/66	97%	53/121	43%
>5;06 (Mean 6;0,12)	60/60	100%	63/110	57%
Total	124/126	98%	116/231	50.2%
**Production**				
5 (Mean 5;05,15)		98%		62.5%

a *The object relatives here include relative clauses with pre- and post-verbal subjects*.

Similar results have been obtained for other languages, such as Italian. In Arosio et al. (2009), which involved 139 Italian-speaking children of ages 5–11, object relatives with post-verbal subjects were miscomprehended at ages 7 and 9 (with adult performance below 50%) and only at 11 was comprehension adult-like (see also Adani, 2010). Parallel results for object wh- interrogatives (also subsumed by Friedmann et al.'s account) showed that 8- to 9-year-olds had not yet achieved adult performance (De Vincenzi et al., 1999; Guasti et al., 2012).

It is beyond the scope of this report to sum up the literature that has been carried out on relative clauses and related constructions over the years, which has led to the development of experiments manipulating Case, number, and gender features (Guasti et al., 2008; Adani et al., 2010; Belletti et al., 2012; Bentea et al., 2016; Friedmann et al., 2017), all relevant to the RM hypothesis^5^. Although Friedmann et al. (2009, p. 71) assert that “the difficulty with object relatives is overcome at around the age of 6 (Friedmann and Novogrodsky, 2004),” the literature on the acquisition of Romance shows that object relatives are not target-like at age 7 (and even beyond) and so, if all of these results are to receive a unified account (a desirable outcome), then we can assume that child RM is operative at age 7, the oldest age group in this study.

To my knowledge, no study so far has considered the child version of RM with grammaticality judgement. The general expectation is that grammaticality judgment should align with production and comprehension, in absence of any indication to the contrary. While dissociations between e.g. production and comprehension are attested in language acquisition, they call for an explanation. If the path of language development is grammar-driven, the prediction is that production, comprehension and judgement will develop in parallel. This is the assumption underlying the experiment in this report. Even though children are known to often fail in their production and comprehension of object relatives in Catalan, and this is attributed to child RM in the literature, they do not judge object relatives in the same way as they judge RM violations. This argues against an assimilation of object relatives and RM violations in child grammar (that is, against the grammatical assimilation of the identity and inclusion conditions).

The results here are exploratory; let us suppose that children do not judge RM violations in the same way they judge object relatives at an age at which the child strict version of RM is operative, as the results so far suggest. In that case, what could the explanation be? Friedmann et al. (2009) do not discard the idea that child RM is the result of a processing limitation. The fact that other populations (language impaired children, patients with aphasia) also perform differently with subject and object relatives, and healthy adults under certain circumstances may also display the same asymmetry (Cohen and Mehler, 1996, and much subsequent work; Warren and Gibson, 2002; see Grillo, 2008) would seem to favor a processing account. In Friedmann et al.'s words (2009, p. 84–85), “It may be tempting to speculate that the ban against [(5b)] in early systems may relate to a limitation in the operative syntactic memory: clearly, disjointness is easier to determine, as it can be calculated feature by feature, whereas calculating a subset-superset relation requires holding in operative memory and comparing the whole featural specifications associated with different positions, an operation that may exceed the capacity of the early systems.” In adults, on the other hand, “a partial overlap of features giving rise to a configuration like [(5b)] is grammatical, but determines ‘complexity effects' detectable in experimental work.” Under such a processing account, one could speculate that the source of the difference between the results here and the results in the literature on relative clause comprehension and production are related to the experimental method. If grammaticality judgement is less costly than comprehension/production in terms of processing (to the extent that the interpretation of the sentence may not need to be fully accessed) then one would predict that object relatives and RM violations would not be judged homogeneously by children, even under the assumption that child RM is operative.

In addition, there is a further difference between object relatives and RM violations, even for children: while children do produce (to varying degrees) object relatives, RM violations of the kind exemplified in (2) and (12) are not attested. This may be an indication that the configuration underlying object relatives is part of child grammar, while RM violations are ungrammatical for children. Grammaticality judgement can therefore provide a new source of evidence to characterize child RM as either a grammatical or a processing phenomenon.

## Data Availability Statement

All datasets generated for this study are freely available at: https://ddd.uab.cat/record/215041.

## Ethics Statement

The studies involving human participants were reviewed and approved by the Comissió d'Ètica en l'Experimentació Animal i Humana (CEEAH), Universitat Autònoma de Barcelona. Written informed consent to participate in this study was provided by the participants' legal guardian/next of kin.

## Author Contributions

AG designed the experiment reported and ran it with the collaboration of Míriam Muntané. AG analyzed the data with the help of Ester Boixadera and wrote the paper.

### Conflict of Interest

The author declares that the research was conducted in the absence of any commercial or financial relationships that could be construed as a potential conflict of interest.

## Footnotes

^1^Intervention is defined structurally (in terms of c-command), not linearly. This was shown clearly in the case of subject and object relative clauses in Chinese, which follow the asymmetry outlined above in a language in which relative clauses are prenominal and therefore linear and structural intervention do not concur (see Hu, 2014).

^2^To be accurate, not all object relatives are problematic for children (for example, in Hebrew object relatives with null pronominal subjects with arbitrary interpretation, as well as free object relatives, do not give rise to intervention effects), and this follows from different featural specifications, i.e., they would not fall under the configuration (5b)—see Friedmann et al. (2009).

^3^The term I use is grammaticality judgement, as is customary in the generative framework, to refer to the task that a speaker performs when asked about the well-formedness or ill-formedness of a sentence (ill-formedness being represented by an asterisk diacritic); acceptability would refer to well-formedness with respect to a given discourse/pragmatic context, which is not at stake. A sentence is standardly assumed to be grammatical when its derivation converges.

^4^The analysis of relative clauses assumed in this literature (and here) is a raising analysis (see Bianchi, 2002a,b).

^5^There is also work disputing the claims of Friedmann et al. (2009) (see, for example, Goodluck, 2010), and some results that the hypothesis cannot readily encompass, especially from studies on topicalization (e.g., Hu et al., 2018 on Chinese)—but this is not discussed in this paper.

## Annex: Experimental Items

Object relatives within transitive clauses (1–6), RM violations (7–12), long-distance interrogatives (13–18):

1.– Veig la nena que la mestra ha renyat.

2.– Veig el gos que la nena buscava.

3.– Tinc el pinzell que els nens buscaven.

4.– Veig el gat que el gos ha mossegat.

5.– Veig els pollets que la gallina buscava.

6.– Tinc el conte que els nens llegiran.

7.– Com dius qui es porta?

8.– Com dius qui vindrà?

9.– Com penses qui vindrà?

10.– Què penses com farà?

11.– Què penses qui arreglarà?

12.– Què penses qui llegirà?

13.– Com dius que ha vingut?

14.– Com dius que va a casa seva?

15.– Què penses que farem avui?

16.– Què penses que farà la senyoreta?

17.– Quan penses que farem vacances?

18.– Quan penses que aniràs a casa?
